# Systematic Evaluation of Imaging Features of Early Bladder Cancer Using Computed Tomography Performed before Pathologic Diagnosis

**DOI:** 10.3390/tomography9050138

**Published:** 2023-09-11

**Authors:** Rubab F. Malik, Renu Berry, Brandyn D. Lau, Kiran R. Busireddy, Prasan Patel, Sunil H. Patel, Elliot K. Fishman, Trinity J. Bivalacqua, Pamela T. Johnson, Farzad Sedaghat

**Affiliations:** 1 The Russell H. Morgan Department of Radiology and Radiological Science, Johns Hopkins University School of Medicine, Baltimore, MD 21287, USArberry@adventisthealthcare.com (R.B.); blau2@jhmi.edu (B.D.L.); kbusire1@jhmi.edu (K.R.B.); ppatel@radiology.ca (P.P.);; 2 Brady Urological Institute, Johns Hopkins University School of Medicine, Baltimore, MD 21287, USA; spate167@jhmi.edu (S.H.P.);

**Keywords:** bladder cancer, urothelial carcinoma, transitional cell carcinoma, opportunistic screening, incidental findings

## Abstract

Background: Bladder cancer is the sixth most common malignancy in the United States (US). Despite its high prevalence and the significant potential benefits of early detection, no reliable, cost-effective screening algorithm exists for asymptomatic patients at risk. Nonetheless, reports of incidentally identified early bladder cancer on CT/MRI scans performed for other indications are emerging in the literature. This represents a new opportunity for early detection, with over 80 million CT scans performed in the US yearly, 40% of which are abdominopelvic CTs. This investigation aims to define the imaging features of early bladder cancer, with the mission of facilitating early diagnosis. Methods: Following IRB approval with a waiver of informed consent, a retrospective review was performed, identifying 624 patients with non-muscle-invasive bladder cancer diagnosed at Johns Hopkins Hospital between 2000 and 2019. Of these patients, 99 patients underwent pelvic CT within the 5 years preceding pathologic diagnosis. These imaging studies were reviewed retrospectively to evaluate for the presence and features of any focal bladder wall abnormality. Results: Median age at the time of pathologic diagnosis was 70 years (range: 51–88 years), and 82% (81/99) of patients were male. A total of 226 CT studies were reviewed. The number of studies per patient ranged from 1 to 33. Median time interval between all available imaging and pathologic diagnosis was 14 months. A total of 62% (141/226) of the scans reviewed were performed for indications other than suspected urinary tract cancer (UTC). A bladder wall mass was visualized in 67% (66/99) of patients and on 35% (78/226) of scans performed before diagnosis. The majority (84%, 67/80) of masses were intraluminal. Mean transverse long- and short-axis measurements were 24 mm and 17 mm, respectively, with long dimension measurements ranging between 5 and 59 mm. Conclusions: Early bladder cancer was visualized on CT preceding pathologic diagnosis in more than 2/3 of patients, and the majority of scans were performed for indications other than suspected urinary tract cancer/UTC symptoms. These results suggest that cross-sectional imaging performed for other indications can serve as a resource for opportunistic bladder cancer screening, particularly in high-risk patients.

## 1. Introduction

Urinary bladder cancer is the sixth most common malignancy in the United States (US), with over 81,000 newly diagnosed cases in 2022. Among males, it is the fourth leading cause of cancer and the eighth leading cause of cancer death [1]. While the incidence is higher in males, females experience worse outcomes [2,3,4,5]. Moreover, bladder cancer imparts significant social cost, constituting the most expensive malignancy to treat per patient over their lifetime [1,6]. Major risk factors for developing bladder cancer include male sex, advanced age, genetic mutations, exposure to arsenic and occupational chemicals, and cigarette smoking, which is responsible for approximately half of the bladder cancer cases [7,8,9,10,11,12,13,14,15,16]. In the past few decades, prevalent cancers such as lung, prostate, breast, and colorectal cancer have seen significant declines in mortality related, in part, to effective screening and early detection protocols [1]. While death rates from bladder cancer have witnessed similar declines in developed regions, disease incidence continues to rise globally [16], and efforts to further improve outcomes have been impeded by the absence of a widely accepted cost-effective screening algorithm in place for asymptomatic individuals who may be at an increased risk of bladder cancer based on genetic predisposition or environmental exposure [13].

The vast majority of urinary bladder cancers are urothelial carcinomas arising from the epithelium (urothelium) lining the inner surface of the urinary bladder. The most critical factor in the pathologic staging of bladder cancer is distinguishing non-muscle-invasive bladder cancer, which exhibits no extension into the lamina propria (NMIBC, ≤T1, 75% of newly diagnosed bladder cancers) from muscle-invasive disease (MIBC, ≥T2a, 25% of newly diagnosed tumors) [8]. While generally considered distinct histopathologic entities, approximately one-half of NMIBC cases can progress to MIBC, thus offering a less favorable prognosis [17,18,19]. The overall 5-year survival rate for individuals diagnosed with bladder cancer is 77% but varies widely depending on the cancer stage at the time of diagnosis, decreasing to 5% for patients presenting with metastatic disease [12]. The distinction between muscle-invasive and non-muscle-invasive bladder cancer is a vitally important prognostic determinant, with significant differences in treatment options and survival. For non-muscle-invasive bladder cancer (NMIBC), first-line treatment involves the transurethral resection of bladder tumor (TURBT) with potential intravesical therapy, while for muscle-invasive bladder cancer (MIBC), treatment is more aggressive and includes a combination of radiation therapy, chemotherapy, and/or radical cystectomy. Hence, early diagnosis of bladder cancer (before the development of muscle invasion) is critical in facilitating less invasive treatment, reducing the rate of recurrence, and improving overall morbidity and mortality [20].

Over 80 million CT scans are performed annually in the United States, of which approximately 40% are abdominopelvic CTs [21]. This represents a significant opportunity for bladder tumor detection, as there is extensive recent literature reporting incidental bladder tumor detection on imaging performed for alternative indications, such as colorectal cancer screening, spinal cord injury, and aortic/peripheral vascular disease [22,23,24,25,26,27,28,29,30,31]. These series illustrate incidentally detected bladder cancer as intraluminal masses or areas of focal wall thickening on cross-sectional imaging [32]. Notably, these lesions are most conspicuous on early contrast-enhanced phases (frequently used in routine imaging) rather than the excretory phase (generally reserved for CT urography) [33].

Based on these reports, we hypothesized that an opportunistic detection of bladder cancer could be improved by elucidating the CT features of these lesions, broadly informing radiologists in study interpretation, protocol optimization, and the development of automated detection algorithms. To achieve this aim, our research investigation systematically defined the morphology of opportunistically detectable bladder lesions, by retrospectively reviewing the characteristic imaging features of early bladder cancer on CT scans performed before a biopsy-confirmed diagnosis, and assessing the time interval between the appearance of a focal bladder abnormality and ultimate pathologic diagnosis.

## 2. Materials and Methods

This retrospective study was approved by the Johns Hopkins Institutional Review Board for Human Research with a waiver of informed consent and complied with Health Insurance Portability and Accountability Act (HIPAA) regulations. From a research registry of six hundred and twenty-four patients with newly diagnosed urothelial carcinoma of the bladder between 2000 and 2019, we identified a cohort of 99 eligible patients with non-muscle-invasive bladder cancer (NMIBC) who had at least one abdominopelvic CT performed within the 5 years preceding their initial biopsy-confirmed diagnosis. Patient charts were reviewed for data collection of the following variables: date of birth, sex, history of cigarette smoking, microscopic and/or gross hematuria, date of pathologic diagnosis of bladder cancer, tumor grade, and imaging history (imaging date, scan type, and clinical indication for all CT of the abdomen/pelvis). Patients included in the cohort had between 1 and 33 available CT studies, all of which were included in subsequent analysis.

**Figure 1:**
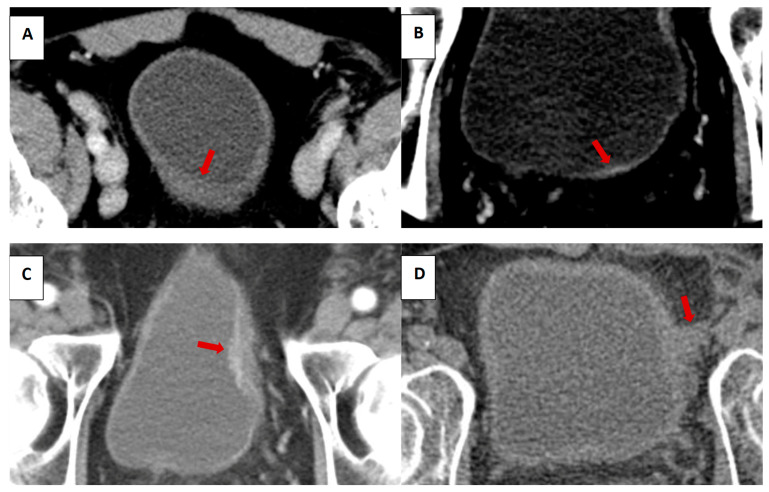
Illustrative examples of focal wall thickening (**A**), focal wall enhancement (**B**), intraluminal mass (**C**), and extramural mass (**D**). Red arrows to indicate bladder lesions.

We assessed the following features on prior CT: IV contrast phases in the pelvis, bladder distention (volume calculated using the prolate ellipsoid method, (length x height x width x 0.57) [34], focal wall thickening (presence and measurement), focal wall enhancement (presence), and the presence and long-axis measurements of the following: intraluminal mass, intramural mass, and extramural mass of the bladder (illustrated in Figure 1). Assessment was made by one of two board-eligible body imaging fellows and confirmed by a board-certified radiologist with over 10 years’ experience. Readers were aware that patients subsequently developed bladder cancer, but were blinded to the date of pathologic diagnosis.

Time elapsed was stratified based on the interval between suspicious imaging findings (i.e., wall thickening, wall enhancement, masses) and pathologic diagnosis. All data were tabulated in Microsoft Excel (Office 365), and descriptive statistics were computed. Mean bladder mass size was computed by finding the mean of the two dimensions.

## 3. Results

Demographics of the 99 patients diagnosed with non-muscle-invasive bladder cancer who had at least one abdominopelvic CT within the 5 years before biopsy-confirmed bladder cancer are detailed in Table 1. Median patient age at the time of diagnosis was 70 years (range: 51–88). A total of 82% (81/99) were male, 66% (65/99) had a history of cigarette smoking, and 79% (78/99) experienced gross hematuria at some point prior to pathologic diagnosis. The distribution of tumor grade on histopathology was: high grade, 77/99 (78%); carcinoma in situ, 15/99 (15%); intermediate grade, 1/99 (1%); mixed high and low grade, 2/99 (2%); and low grade, 4/99 (4%).

Between 1 and 33 imaging studies were reviewed per patient for a total of 226 CT performed before pathologic diagnosis. The median time interval from imaging to pathologic diagnosis was 14 months (range: 1 day–58 months). Of the 226 scans reviewed, 141 (62%) imaging studies were performed for alternative indications not related to suspected urinary tract cancer symptoms. Nineteen patients (19%) had a bladder tumor present on prior imaging and also had multiple imaging studies in their medical chart, which were reviewed in this study. Among these 19 patients, bladder tumors were visualized on one imaging study in 74% (14/19) and on multiple prior imaging studies in 26% (5/19).

**Figure 2:**
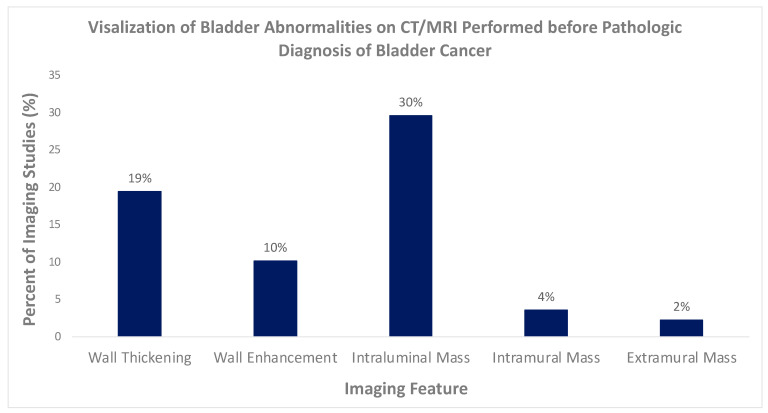
Visualization of bladder abnormalities on CT performed before pathologic diagnosis of bladder cancer.

Figure 2 demonstrates the overall rate of abnormal bladder findings. Bladder wall thickening, wall enhancement, intraluminal mass, intramural mass, and extramural mass were visualized in 19%, 10%, 30%, 4%, and 2% scans, respectively, on scans performed during the 5 years preceding biopsy-confirmed diagnosis. A bladder wall mass was visualized in 67% (66/99) of patients and on 35% (78/226) of scans. Bladder masses ranged from 5 to 59 mm, with a mean size of 24 mm. The majority of the visualized masses were intraluminal (67/80, 84%), while (8/80, 10%) were intramural and (5/80, 6%) were extramural.

Table 2 demonstrates the temporality of the visualization of bladder masses (i.e., intraluminal, intramural, extramural). In a small percentage of cases, an intraluminal mass was identified retrospectively on remote exams (4% of scans performed greater than 3 years before pathologic diagnosis and 4% for scans within 12–18 months of diagnosis). This increased to 21% for scans within 6–12 months and 63% of scans were performed within 6 months of diagnosis.

**Figure 3:**
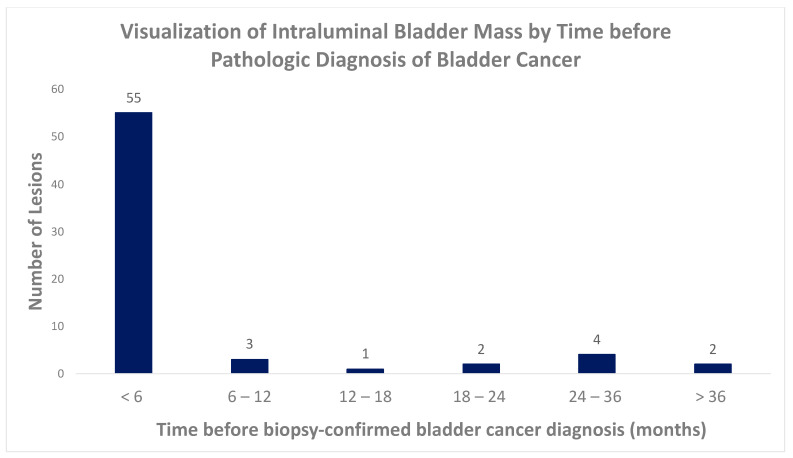
Visualization of intraluminal bladder mass by time before pathologic diagnosis of bladder cancer.

**Figure 4:**
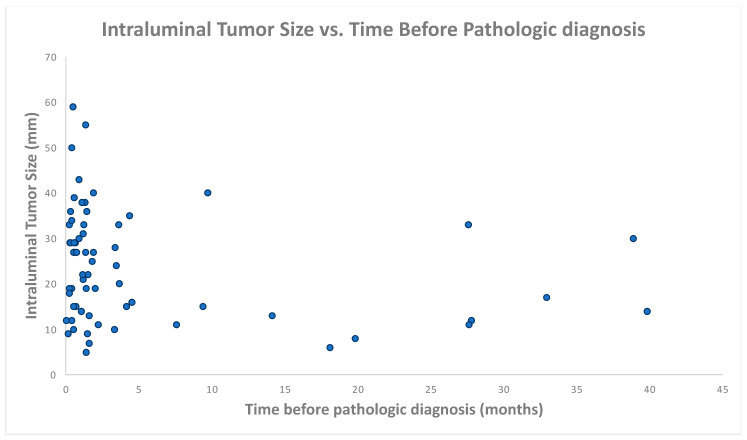
Intraluminal tumor size vs. time before pathologic diagnosis.

Figure 3 illustrates the temporal relationship between the visualization of intraluminal bladder masses on CT and pathologic diagnosis. The majority of intraluminal masses (55/67, 82%) were visualized on imaging performed within 6 months before diagnosis; 6% (4/67) of intraluminal masses were visualized 2–3 years before diagnosis, and 3% (2/67) of intraluminal masses were visualized more than 3 years before pathologic diagnosis. Figure 4 illustrates the tumor size in millimeters of intraluminal tumors as a function of time before pathologic diagnosis.

The most commonly used scanning protocols were non-contrast CT abdomen and pelvis (*n* = 30, 12.7%), portal venous phase abdomen and pelvis (*n* = 76, 32.3%), and multiphasic CT urogram (*n* = 99, 42.1%), with lesion detection rates measuring 26.8%, 15.6%, and 33.1%, respectively. Mean bladder volumes on non-contrast and portal venous phase exams with detectable tumors were 390.3 mL and 210.7 mL, respectively, compared to volumes of 184.9 mL and 202.8 mL on exams without detectable tumors. The mean size of tumors detected on non-contrast and portal venous phase exams were 23 mm and 21 mm, respectively (Table 3). With respect to scan indication, 48% of exams were performed for oncologic surveillance (excluding UTC). Notably, none of the portal venous phase exams were performed for urinary tract cancer symptoms.

## 4. Discussion

**Figure 5:**
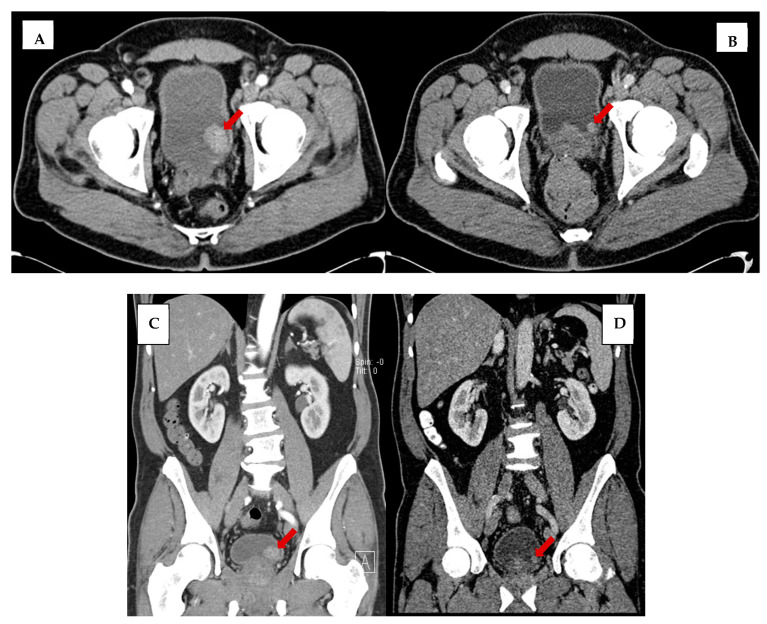
Axial view of bladder tumor (**A**) at time of bladder cancer diagnosis and (**B**) 2 years before diagnosis on imaging performed for diverticulosis. Coronal view of bladder tumor (**C**) at time of diagnosis and (**D**) 2 years before diagnosis. Red arrows to indicate bladder mass. Note the increased noise in images (**B**,**D**) secondary to dose reduction techniques.

In a retrospective review of abdominopelvic CT imaging performed before a pathologically established diagnosis of non-muscle-invasive bladder cancer, we found that a bladder mass was discernable in over two-thirds of the patients, and in nearly one-third of the CT studies performed up to five years preceding the biopsy-confirmed diagnosis. Figure 5 is an illustration of one such example in a 62-year-old male who presented with left lower quadrant pain and hematuria and was worked up for bladder cancer with a CT of the abdomen and pelvis that showed a 2.6 cm intraluminal bladder tumor. In retrospect, a 1.0 cm bladder mass was present on a CT performed 2 years prior for diverticular disease. This highlights the need for the careful inspection of the urinary bladder wall in all abdominopelvic CT performed for any indication, and not merely those dedicated to evaluation of urinary tract symptoms.

In our study, only patients with non-muscle-invasive bladder cancer were included to highlight the characteristic imaging features of early-stage bladder cancer and facilitate early diagnosis. Notably, of the 67 intraluminal non-muscle-invasive bladder cancers visualized on imaging studies preceding pathologic diagnosis, 9% (6/67) were present 2 or more years before a diagnosis (Figure 3) on retrospective review. Small lesions are admittedly easier to appreciate in retrospect with a subsequent study for comparison, so the prospective sensitivity in real world practice may be lower.

The primary objective of our study was to assess the potential for opportunistic tumor detection. As such, the results of pelvic portal venous phase exams are the most salient finding, as this phase of imaging is limited to the abdomen in our institution’s genitourinary cancer screening protocol, with only the arterial and delayed phases including the abdomen and pelvis. Given the prevalence of bladder cancer and the widespread usage of portal venous CT imaging, our results suggest that a significant number of patients could benefit from incidental tumor detection, despite the relative insensitivity of this technique (15.6% detection rate). Notably, there was no significant difference in bladder distention on portal venous phase exams with and without detected tumor (210.7 mL vs. 202.8 mL), as opposed to non-contrast exams where greater bladder distention was linked to increased tumor detection (390.3 mL vs. 184.9 mL). This result suggests the potential for optimizing non-contrast exams to improve incidental bladder mass detection, via hydration and a period of non-voiding.

Given these findings, a routine inspection of the bladder with a high-contrast window and multiplanar reconstructions should be a part of the radiologist’s search pattern, particularly in patients over age 50, like our cohort. Only scrutinizing patients with known exposures, such as smoking, may exclude at-risk patients, as studies have demonstrated that a significant proportion of patients with UTC may have elevated amylamine levels (the leading putative cause of bladder cancer among smokers), despite having no history of tobacco abuse [35]. Also notable is the high proportion of patients undergoing surveillance for malignancies other than UTC within our cohort (48%), suggesting that specific attention should be provided to this patient population.

Significantly, a comparison of the relative performance of non-contrast, portal venous, and CT urogram techniques in our study is not meaningful, as the evaluated patient populations differ dramatically. Expectedly, the lowest detection rate was in patients undergoing portal venous phase exams, as presumably none of these patients had acute genitourinary symptoms. Conversely non-contrast CT is commonly performed for nephrolithiasis and hematuria (in patients with significant renal impairment), and CT urogram is the standard technique for the assessment of hematuria and urothelial masses. The difference in patient populations is corroborated by the smaller mean tumor size in patients undergoing portal venous phase exams (21 mm compared to a population mean of 24 mm), which is an expected finding, as these patients were asymptomatic, and their tumors incidentally detected.

Although our results suggest that a meaningful proportion of asymptomatic bladder cancers may be incidentally detected, the issue of specificity, and the possibility of false positives, was not addressed by our investigation. This is a notable limitation, as tumor detection can be significantly confounded by bladder wall trabeculation [36,37], a finding commonly seen in elderly men due to BPH/urinary retention. This leaves radiologists with a clinical dilemma; not only is this the demographic with the highest incidence of bladder cancer, but there is also a link between BPH and the subsequent development of bladder cancer [1,38,39]. Moreover, while we presume patients undergoing portal venous phase CT were not experiencing genitourinary symptoms (as this would warrant a multiphasic CT urogram), many of the examinations were a part of routine oncologic surveillance, and patients may not have undergone extensive clinical or laboratory evaluation (hence, clinical symptoms may have escaped unrecognized). Given these challenges, future investigations using a control group of patients without bladder cancer are needed to elucidate specific clinical and radiologic features that warrant further assessment. Moreover, while the presence of potential tumor mimics the general screening with portal venous phase CT, and the relatively low detection rate on portal venous scans suggests that this would likely be ineffective, a prospective study would be necessary to confirm. In addition, while thin slice imaging was generally available, variations in dose and scanner were not uncommon, which may have yielded differences in imaging quality that are beyond the scope of our investigation. An additional limitation is the retrospective nature of the investigation, which may have spuriously increased the sensitivity.

Another area of potential inquiry is artificial intelligence (AI). Bladder cancer, as a highly prevalent malignancy with no routine screening, could be a prime target for emerging AI techniques. While tumor was retrospectively detectable in some patients, these patients were the minority, with opportunistic detection hampered by low exam sensitivity and specificity. Emerging deep learning AI technologies may help address these issues, with computer-assisted detection (CADe) aiding in the detection of subtle lesions (increasing sensitivity) and computer-assisted diagnosis (CADx) discriminating between benign findings and malignancy (increasing specificity) [40]. While AI has been successfully applied in the segmentation of known bladder tumors and the monitoring of treatment response [41], prospective investigations using AI for incidental bladder tumor detection and lesion characterization remain limited, warranting further investigation [42,43].

To our knowledge, our investigation represents the first systematic review of the imaging features of early bladder cancer and their temporal relationship to a histopathologic diagnosis of UTC. We share the results of our retrospective observational study, aiming to increase awareness among radiologists of the early imaging features of bladder cancer and the possibility of incidental tumor detection. By emphasizing the benefits of the early opportunistic detection of NMIBC, we hope to spearhead a potential cost-effective screening method for bladder cancer. We recognize our study has significant limitations. As a single-site retrospective investigation, our results may not be generalizable to other medical institutions/patient populations, warranting future multicenter studies. Furthermore, there is inherent bias resulting from the focused assessment of the urinary bladder in pathologically confirmed UTC cases, a scenario not reflected in everyday practice. Additionally, assessing the predictive value of the suspicious imaging features and delineating tumor mimics was beyond the scope of our investigation, and should be assessed in future research. Despite these limitations, we hope our investigation increases mindful awareness of bladder cancer, and its staggering morbidity/mortality, helping to reset the threshold for the reporting of subtle bladder findings and leading to further technique optimization, potentially including hydration, and non-voiding prior to non-contrast CT exams.

## 5. Conclusions

As no standardized screening algorithm for bladder cancer detection currently exists, systematic opportunistic screening for bladder abnormalities would represent a paradigm shift among radiologists, potentially facilitating early diagnosis [44]. We believe that during abdominopelvic imaging performed for alternative indications, a concentrated bladder survey could significantly aid in bladder cancer detection, particularly in high-risk patients (those over 50 years of age, especially patients with a history of tobacco abuse and/or industrial chemical exposures). Early asymptomatic detection could, in turn, decrease morbidity and increase cancer survival [45], improving the quality of life for patients through timely intervention, and the full utilization of minimally invasive treatment options.

## Figures and Tables

**Table 1:** Summary of patient cohort and imaging studies.

Patient Cohort (*n* = 99)	
Age, median (range)	70 (51–88)
Male sex, no. (%)	81 (82%)
History of gross hematuria, no. (%)	78 (79%)
History of cigarette smoking, no. (%)	65 (66%)
**Imaging Studies (*n* = 226)**	
No. of imaging studies reviewed per patient, median (range)	1 (1–33)
Time interval between imaging and biopsy-confirmed bladder cancer diagnosis, median (range)	14 months (1 day–58 months)

**Table 2:** Visualization of bladder abnormalities on imaging studies by time before pathologic diagnosis.

Time before Pathologic Diagnosis (Months)	No. of Imaging Studies	Wall Thickening	Wall Enhancement	Intraluminal Mass	Intramural Mass	Extramural Mass
0–6	88	20% (18/88)	14% (12/88)	63% (55/88)	6% (5/88)	5% (4/88)
6–12	14	29% (4/14)	14% (2/14)	21% (3/14)	0%	0%
12–18	23	26% (6/23)	17% (4/23)	4% (1/23)	4% (1/23)	0%
18–24	13	46% (6/13)	15% (2/13)	15% (2/13)	15% (2/13)	0%
24–36	31	26% (8/31)	10% (3/31)	13% (4/31)	0%	0%
>36	57	4% (2/57)	0%	4% (2/57)	0%	2% (1/57)
Total	226	19% (44/226)	10% (23/226)	30% 67 (226)	4% (8/226)	2% (5/226)

**Table 3:** Incidentally detected NMIBC on portal venous phase CT.

Portal Venous Phase Exams (*n* = 90)	
Detection rate	16% (14/90)
Mean tumor size	21 mm
Mean bladder vol., exams w/detectable tumor	211 mL
Mean bladder vol., exams w/o detectable tumor	203 mL

## Data Availability

The data presented in this study are not publicly available due to HIPAA privacy restrictions, IRB data sharing restrictions.
